# Characterizing standard genetic parts and establishing common principles for engineering legume and cereal roots

**DOI:** 10.1111/pbi.13135

**Published:** 2019-05-23

**Authors:** Doreen Feike, Andrey V. Korolev, Eleni Soumpourou, Eiichi Murakami, Dugald Reid, Andrew Breakspear, Christian Rogers, Simona Radutoiu, Jens Stougaard, Wendy A. Harwood, Giles E. D. Oldroyd, J. Benjamin Miller

**Affiliations:** ^1^ John Innes Centre Norwich Research Park Norwich UK; ^2^ Department of Molecular Biology and Genetics Aarhus University Aarhus Denmark; ^3^ School of Biological Sciences University of East Anglia, Norwich Research Park Norwich UK; ^4^ Present address: EMBL Heidelberg Meyerhofstraße 1 69117 Heidelberg Germany; ^5^ Present address: Sainsbury Laboratory University of Cambridge 47 Bateman Street Cambridge CB2 1LR UK; ^6^ Present address: GRA&GREEN Inc., Incubation Center 106 Nagoya University Furo‐cho, Chikusa‐ku Nagoya 464‐0814 Japan

**Keywords:** plant synthetic biology, cereal engineering, promoter, terminator, codon optimization, intron‐mediated enhancement

## Abstract

Plant synthetic biology and cereal engineering depend on the controlled expression of transgenes of interest. Most engineering in plant species to date has relied heavily on the use of a few, well‐established constitutive promoters to achieve high levels of expression; however, the levels of transgene expression can also be influenced by the use of codon optimization, intron‐mediated enhancement and varying terminator sequences. Most of these alternative approaches for regulating transgene expression have only been tested in small‐scale experiments, typically testing a single gene of interest. It is therefore difficult to interpret the relative importance of these approaches and to design engineering strategies that are likely to succeed in different plant species, particularly if engineering multigenic traits where the expression of each transgene needs to be precisely regulated. Here, we present data on the characterization of 46 promoters and 10 terminators in *Medicago truncatula*,* Lotus japonicus*,* Nicotiana benthamiana* and *Hordeum vulgare*, as well as the effects of codon optimization and intron‐mediated enhancement on the expression of two transgenes in *H. vulgare*. We have identified a core set of promoters and terminators of relevance to researchers engineering novel traits in plant roots. In addition, we have shown that combining codon optimization and intron‐mediated enhancement increases transgene expression and protein levels in barley. Based on our study, we recommend a core set of promoters and terminators for broad use and also propose a general set of principles and guidelines for those engineering cereal species.

## Introduction

Plant synthetic biology seeks to engineer novel traits into plants, and these engineering efforts need to be specifically directed in crop species to be of agronomic relevance (Kotopka *et al*., 2018; Liu and Stewart, 2015). The engineering of cereals in particular offers up a unique set of challenges, especially as many of the existing tools for plant synthetic biology have been developed for model dicotyledonous plant species. For example, the number of different promoter sequences used in cereal engineering is relatively low and relies heavily on the repeated use of well‐established constitutive promoters such as the 35S, maize ubiquitin or rice actin promoters (Himmelbach *et al*., 2007; McElroy and Brettell, 1994). Engineering of complex traits requires the expression of multiple stacked transgenes, and the repetitive use of genetic parts (e.g. promoters and terminators) is not desirable due to potential problems of T‐DNA stability/integrity and gene silencing in future generations (Meyer and Saedler, 1996). Furthermore, such complex traits may also require precise control and accurate regulation of transgene expression, for example at the level of individual cells and tissues, and at particular times during development. At present, the number of genetic parts for plant synthetic biology and particularly cereal engineering is limited, and there is need to expand the number of characterized standard genetic parts to realize the opportunities in plant engineering (Schaumberg *et al*., 2016).

The choice of promoter sequence is the most common factor when considering the control and regulation of transgene expression levels in plants, and considerable research to date has focussed on identifying promoter regions which are functional and which give desirable expression patterns. Previous studies on promoter characterization have typically been small scale, with promoters often tested individually or in small groups (Park *et al*., 2010); very few large‐scale side‐by‐side comparisons have been made to date. This therefore makes it difficult to interpret the relative usefulness of each promoter sequence for cereal engineering. Furthermore, the development of promoters which can be reliably used across different plant species is of particular interest to plant synthetic biology (e.g. to allow testing of constructs in dicotyledonous plants before embarking on the more time‐consuming generation of transgenic cereals), but this again has not been fully explored to date. Previous research has also demonstrated that introns can enhance transgene expression levels in plants, for example addition of introns in the 5′ untranslated region of transgenes (Jeong *et al*., 2006; Karthikeyan *et al*., 2009; Mitsuhara *et al*., 1996) or within the coding sequence (Bartlett *et al*., 2009; Rose, 2004). Codon optimization (Webster *et al*., 2017) and the use of different terminator sequences (Ingelbrecht *et al*., 1989; Nagaya *et al*., 2010) have also been shown to influence transgene expression. The relative importance of these different factors for governing transgene expression has not been fully explored in a systematic approach, and it is therefore difficult to know which of these are most useful or appropriate. Recent advances in cloning technologies, for example modular Golden Gate cloning methods (Patron *et al*., 2015; Weber *et al*., 2011), have facilitated rapid and easy creation of multigene constructs. Such modular cloning technologies allow complete selection of all genetic parts within a construct, and the above issues about genetic part selection and engineering strategies have now become even more important for cereal engineering.

In this work, we have characterized a library of modular genetic parts with the intent of optimizing transgene expression for the purpose of engineering symbiotic nitrogen fixation into cereal roots. However, within our studies we have included characterization of these standard genetic parts in both dicotyledonous and monocotyledonous plants. We have in particular defined promoters for engineering the roots of cereals, but we have also identified specific promoters that can be used to drive transgene expression across different plant species. Our comparisons of codon optimization and intron‐mediated enhancement also demonstrate the importance of these approaches to control transgene expression in cereal roots independently of promoter/terminator sequences. These studies provide insights into optimal approaches for cereal engineering and facilitate the production of core sets of promoters and terminators for general use in plant synthetic biology.

## Results and discussion

### Defining a library of standard genetic parts of promoters and terminators for cereal engineering

A survey of existing literature was performed to identify promoter sequences (defined as combined upstream regulatory regions, core promoters and 5′ untranslated regions) which we considered useful candidates for plant synthetic biology and specifically for engineering cereal species. This allowed us to define a library of standard genetic parts consisting of 46 different promoter sequences, and these were classified according to three different attributes relevant to the needs of our engineering project and of the cereal engineering community in general: constitutive promoters, symbiosis‐related promoters and root‐specific promoters. Promoters from both dicotyledonous and monocotyledonous plants were selected for each of these three subgroups. The decision to include each promoter sequence was based on the original publications reporting the initial characterization of each individual genetic part (the designation of a ‘constitutive’ or ‘root‐specific’ promoter was based on the original publication rather than our own analysis). Defined promoter sequences were available for most of these individual genetic parts (see File S1 for full details, including references and promoter sequence information); however, we needed to define promoter sequences for many of the symbiosis‐related genes, particularly for cereal species. We focussed on two genes essential for symbiosis signalling: *CCaMK*, a calcium‐ and calcium/calmodulin‐dependent protein kinase (Levy *et al*., 2004; Mitra *et al*., 2004) and the transcription factor *CYCLOPS* (Messinese *et al*., 2007; Yano *et al*., 2008). Using BLAST analyses, we identified homologs of *CCaMK* from *Setaria italica*,* Brachypodium distachyon*,* Zea mays*,* Sorghum bicolor* and *Oryza sativa* and homologs of *CYCLOPS* from *S. italica* and *O. sativa*. The promoter sequence for each of these cereal symbiosis genes was subsequently selected as the upstream region from the start codon of the gene until the start/stop codon of the preceding annotated/predicted open reading frame (see File S1 for sequence information).

In addition to the 46 promoters, we also selected 10 terminator sequences (defined as combined 3′ untranslated regions and transcribed terminators) for inclusion in our library of standard genetic parts (see File S1). Most of these terminators have been widely tested in dicotyledonous species, for example the model plant *Arabidopsis thaliana*; however, the function of many of these terminators has not been tested in cereal species, and we therefore considered it essential to include these in our analysis. All promoters and terminators were cloned into appropriate Golden Gate cloning vectors and conformed to the common syntax described by Patron *et al*. (2015) (see Experimental Procedures).

### Characterization of our library of standard genetic parts: promoter‐GUS testing in dicotyledonous species

**Figure 1:**
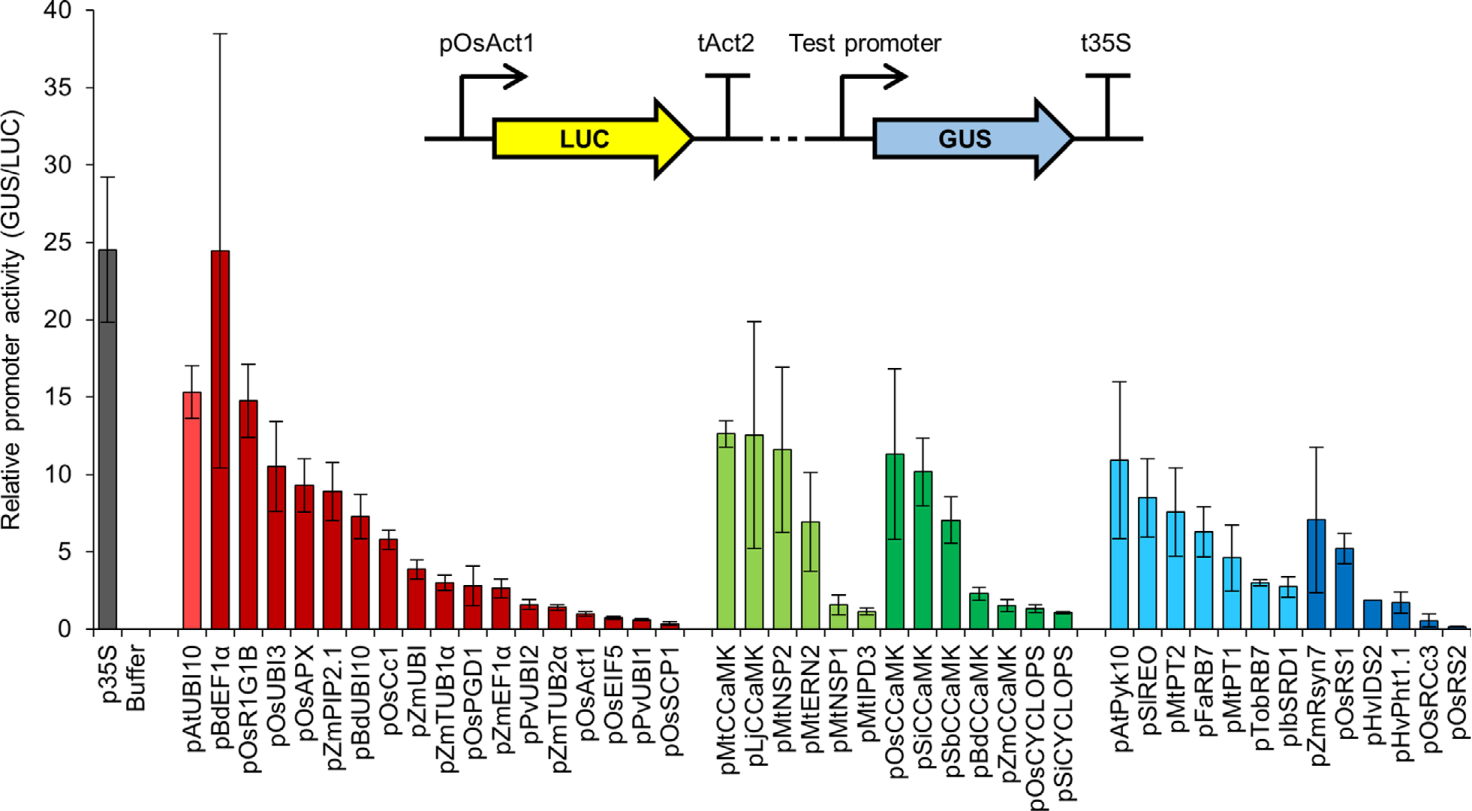
Different constitutive, symbiosis‐related and root‐specific promoters from the standard genetic parts library show varying levels of activity in *Nicotiana benthamiana*. Multigene constructs containing promoters from the standard genetic parts library driving expression of the β‐glucuronidase (*GUS*) reporter gene, plus a constitutively expressed firefly luciferase gene (*LUC*; see figure inset), were transiently expressed in *N. benthamiana* via *Agrobacterium tumefaciens*‐mediated transformation. GUS and LUC reporter gene activities were quantified using a plate reader, and relative promoter activity was calculated by determining the GUS to LUC ratio (with normalization to the pOsAct1 promoter, set at 1). Data represent mean ± standard error from three independent biological replicates. Dotted line in figure inset represents 2.5 kb of plasmid sequence that was unchanged between constructs containing different test promoters. Colour coding: control p35S promoter, grey; constitutive promoters, red; symbiosis‐related promoters, green; root‐specific promoters, blue; light and dark shades denote promoters from dicotyledonous and monocotyledonous species, respectively.

Transient expression of genes in *Nicotiana benthamiana* via *Agrobacterium tumefaciens*‐mediated transformation has been used extensively for rapid, high‐throughput screening. We therefore initially characterized the behaviour of the different promoters from our genetic parts library in *N. benthamiana* using constructs containing each promoter driving the expression of the β‐glucuronidase (*GUS*) reporter gene. To determine relative promoter activity, we quantified the level of GUS enzymatic activity with a 4‐methylumbelliferyl‐β‐d‐glucuronide (MUG) fluorometric assay, using a constitutively expressed firefly luciferase (*LUC*) reporter gene as an internal normalization control for transformation efficiency (Figure 1). This quantitative analysis identified constitutive, symbiosis‐related and root‐specific promoters with different expression levels in *N. benthamiana* leaves and allowed us to categorize each promoter within these subgroups as displaying either high, medium or low relative activity (Table 1; File S2). Promoters from all three subgroups showed a range of activities, and there was no obvious difference in performance between promoters selected from dicotyledonous or monocotyledonous plant species (Figure 1). The highest expression levels were observed with the constitutive promoters p35S, pAtUBI10, pBdEF1α, pOsR1G1B and pOsUBI3; the symbiosis‐related promoters pMtCCaMK, pLjCCaMK, pMtNSP2, pOsCCaMK and pSiCCaMK; and the root‐specific promoter pAtPyk10 (Figure 1). The quantitative nature of our analysis additionally allows for direct comparisons to be made between different promoters; for example, the constitutive promoters pAtUBI10 and pOsR1G1B had similar activity to the symbiosis‐related promoter pMtCCaMK, and all three of these high activity promoters displayed approximately twice the activity of the medium activity promoters pBdUBI10, pOsCc1 and pFaRB7 (Figure 1; Table 1; File S2).

**Table 1:** Summary of promoters tested in this study

Promoter name	Leaf	Root	Cereal	Category
pBdEF1α	HIGH	HIGH	HIGH	1
p35S	HIGH	HIGH	MED	1
pAtUBI10	HIGH	HIGH	MED	1
pOsUBI3	HIGH	HIGH	MED	1
pZmUBI	MED	HIGH	HIGH	1
pBdUBI10	MED	MED	HIGH	1
pOsPGD1	MED	MED	MED	1
pOsR1G1B	HIGH	HIGH	LOW	2
pMtCCaMK	HIGH	HIGH	LOW	2
pLjCCaMK	HIGH	HIGH	LOW	2
pOsCCaMK	HIGH	HIGH	LOW	2
pSiCCaMK	HIGH	HIGH	LOW	2
pMtPT2	HIGH	HIGH	LOW	2
pOsAPX	HIGH	MED	LOW	2
pZmPIP2.1	HIGH	MED	LOW	2
pAtPyk10	HIGH	MED	LOW	2
pSlREO	HIGH	MED	LOW	2
pMtNSP2	HIGH	–	LOW	2
pFaRB7	MED	HIGH	LOW	2
pTobRB7	MED	HIGH	LOW	2
pSbCCaMK	MED	HIGH	LOW	2
pOsCc1	MED	HIGH	LOW	2
pZmTUB1α	MED	MED	LOW	2
pMtPT1	MED	MED	LOW	2
pIbSRD1	MED	MED	LOW	2
pZmRsyn7	MED	MED	LOW	2
pOsRS1	MED	MED	LOW	2
pPvUBI2	LOW	LOW	HIGH	3
pPvUBI1	LOW	LOW	HIGH	3
pOsAct1	LOW	LOW	MED	3
pOsRS2	LOW	MED	MED	4
pOsSCP1	LOW	MED	LOW	5
pMtIPD3	LOW	MED	LOW	5
pZmCCaMK	LOW	MED	LOW	5
pOsCYCLOPS	LOW	MED	LOW	5
pHvIDS2	LOW	MED	LOW	5
pHvPht1.1	LOW	MED	LOW	5
pOsRCc3	LOW	MED	LOW	5
pZmEF1α	MED	LOW	LOW	6
pBdCCaMK	MED	LOW	LOW	6
pMtERN2	MED	–	LOW	6
pZmTUB2α	LOW	LOW	LOW	7
pOsEIF5	LOW	LOW	LOW	7
pSiCYCLOPS	LOW	LOW	LOW	7
pMtNSP1	LOW	–	LOW	7
pHvPht1.2	–	LOW	LOW	7

Promoter activity was scored according to results from *Nicotiana benthamiana* leaves (leaf), *Medicago truncatula* and *Lotus japonicus* roots (root) and *Hordeum vulgare* roots (cereal). High (green), medium (amber) or low (red) scoring is based on promoter activity being ranked either in the top, middle or bottom third of all tested promoters. The assigned categories are as follows: 1, good for leaves and roots in both monocotyledonous and dicotyledonous species; 2, good for leaves and roots in dicotyledonous species, but not best for cereal roots; 3, better for cereal roots than roots or leaves from dicotyledonous species; 4, generally better for roots, but not ideal for leaves; 5, better for roots of dicotyledonous species, but not ideal for leaves or cereal roots; 6, better for leaves of dicotyledonous species, but not ideal for roots; 7, least good promoters. Hyphen denotes untested experimental condition. Also see File S2 for a full summary of all of the characterization data presented in this work.

**Figure 2:**
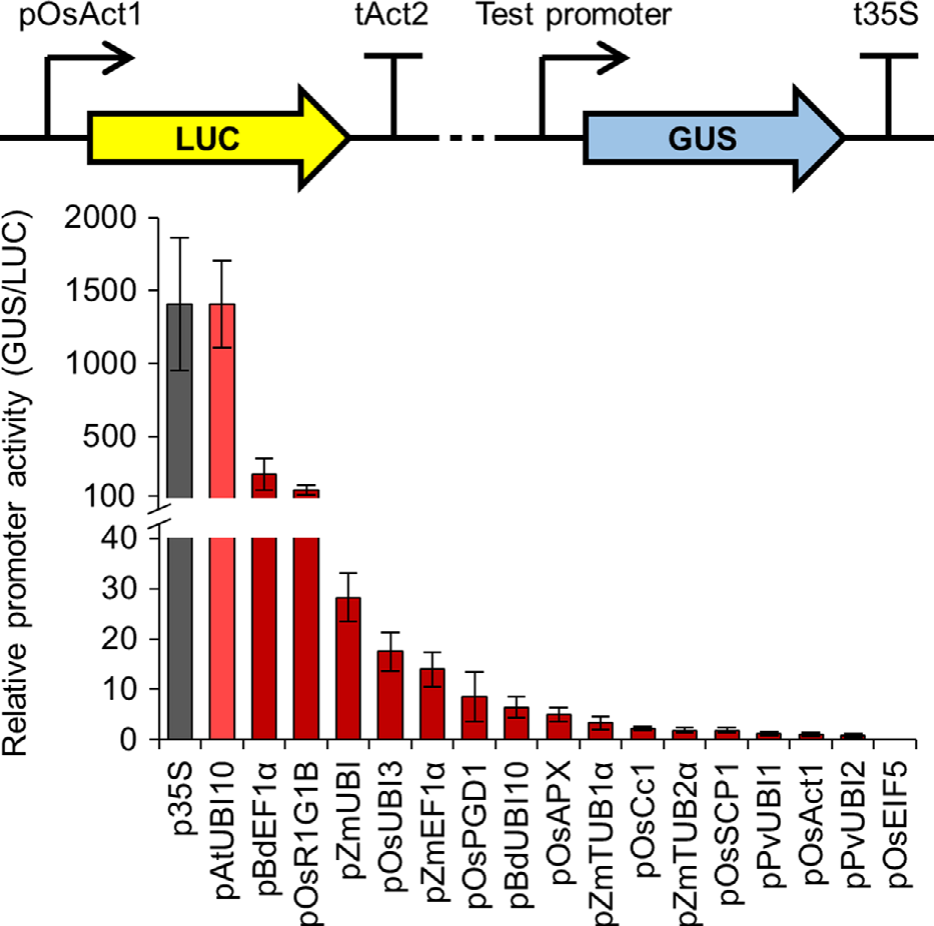
Different constitutive promoters from the standard genetic parts library show varying levels of activity in *Medicago truncatula*. Multigene constructs containing promoters from the standard genetic parts library driving expression of the β‐glucuronidase (*GUS*) reporter gene, plus a constitutively expressed firefly luciferase gene (*LUC*; see figure inset), were expressed in *M. truncatula* via *Agrobacterium rhizogenes*‐mediated transformation. GUS and LUC reporter gene activities were quantified using a plate reader, and relative promoter activity was calculated by determining the GUS to LUC ratio (with normalization to the pOsAct1 promoter, set at 1). Data represent mean ± standard error from three independent biological replicates. Dotted line in figure inset represents 2.5 kb of plasmid sequence that was unchanged between constructs containing different test promoters. Colour coding: control p35S promoter, grey; constitutive promoters, red; light and dark shades denote promoters from dicotyledonous and monocotyledonous species, respectively. No *GUS* expression was observed with the pOsEIF5 promoter.

Since our engineering is focussed on roots, we tested the expression of each promoter using the model legume *Medicago truncatula*, where we can rapidly generate many plants with transgenic roots via *Agrobacterium rhizogenes*‐mediated transformation (Boisson‐Dernier *et al*., 2001). We used the same constructs as for *N. benthamiana* and performed quantitative MUG fluorometric assays using the constitutively expressed firefly *LUC* reporter gene as an internal normalization control for transformation efficiency. We only tested the constitutive promoters subgroup in *M. truncatula* and found that all (except pOsEIF5) were expressed in root tissue (Figure 2). Although the values of relative promoter activity varied between *M. truncatula* roots and *N. benthamiana* leaves, the overall correlation of promoter activity in *M. truncatula* roots closely matched that in *N. benthamiana* leaves (Figure 1 and Figure S1a), suggesting that tissue type does not strongly influence the relative expression level of the constitutive promoters in these two different dicotyledonous species. For example, the p35S and pAtUBI10 promoters had the highest levels of expression in *M. truncatula*, followed by pBdEF1α and pOsR1G1B (Figure 2); these four promoters also showed the highest expression levels in *N. benthamiana* (Figure 1). We therefore particularly recommend these four promoters as the best for achieving high levels of transgene expression in dicotyledonous plant species (Table 1; File S2). A number of the constitutive promoters had very low levels of expression in *M. truncatula* roots (e.g. pZmTUB2α, pPvUBI1, pOsAct1 and pPvUBI2; Figure 2), and these promoters showed similarly low expression levels in *N. benthamiana* (Figure 1 and Figure S1a). We therefore categorize these promoters as having low activity and would not recommend them for achieving the highest levels of expression in *N. benthamiana* leaves or *M. truncatula* roots (Table 1; File S2).

### Characterization of promoters for symbiosis signalling in *Medicago truncatula* and *Lotus japonicus*

**Figure 3:**
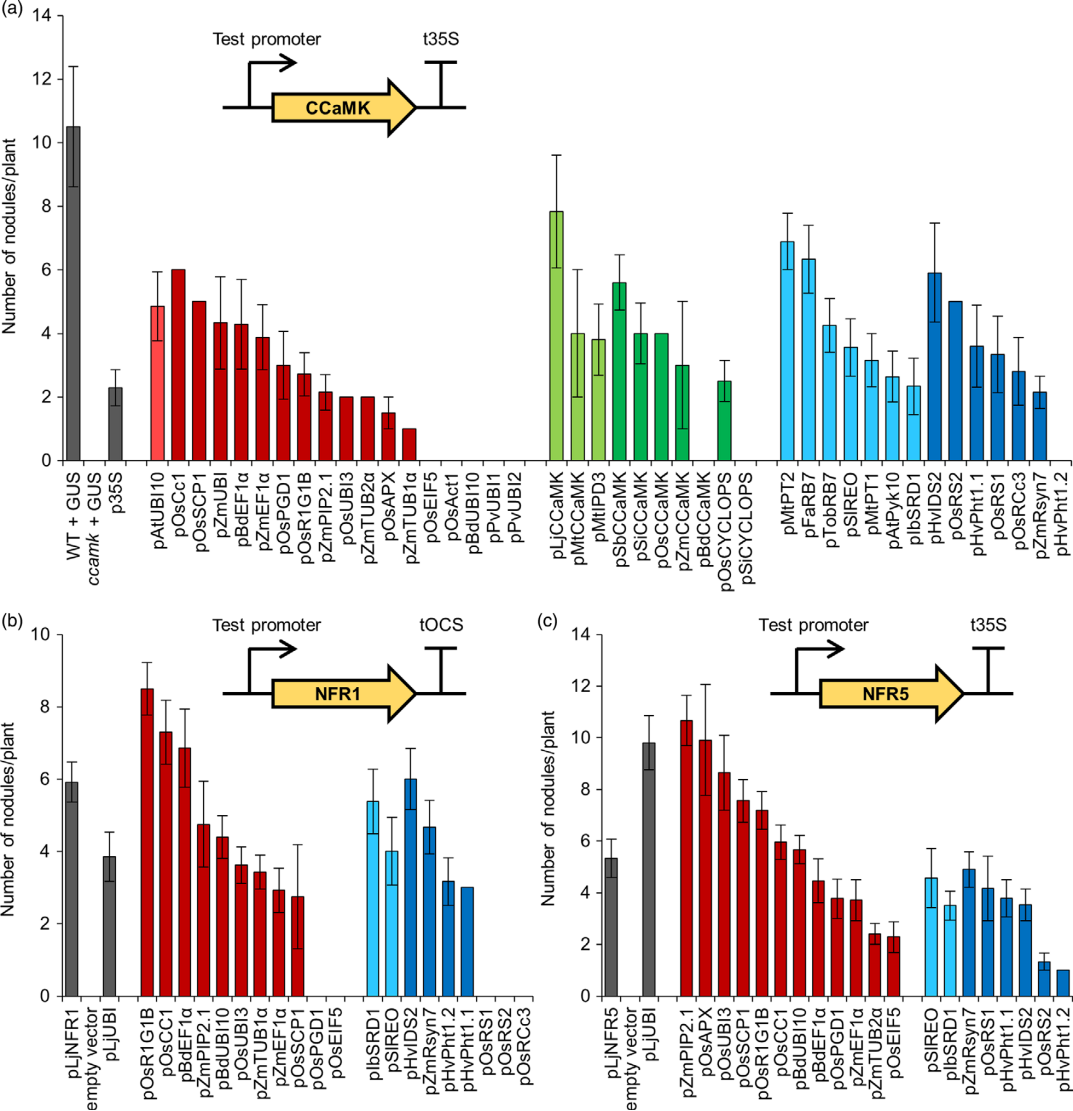
Different constitutive, symbiosis‐related and root‐specific promoters from the standard genetic parts library show varying abilities to complement the *ccamk* mutant of *Medicago truncatula* and the *nfr1* and *nfr5* mutants of *Lotus japonicus*. Constructs containing promoters from the standard genetic parts library driving expression of the *CCaMK* (a), NFR1 (b) or NFR5 (c) genes (see figure insets) were transformed into *Medicago truncatula ccamk‐1* (a), *Lotus japonicus nfr1‐1* (b) or *nfr5‐2* (c) mutant plants via *Agrobacterium rhizogenes*‐mediated transformation. Total root nodule number was counted three weeks after inoculation with *Sinorhizobium meliloti* in *M. truncatula*, or five weeks after inoculation with *Mesorhizobium loti* in *L. japonicus*. Data represent mean ± standard error. In total, an average of at least 10 independently transformed plants was scored per construct (dsRED in the T‐DNA confirmed transformation). WT, wild type. Colour coding: control p35S and pLjUBI promoters, native pLjNFR1 and pLjNFR5 promoters, and control plants transformed with pAtUBI10‐GUS construct (GUS) or empty vector, grey; constitutive promoters, red; symbiosis‐related promoters, green; root‐specific promoters, blue; light and dark shades denote promoters from dicotyledonous and monocotyledonous species, respectively. Promoters pOsEIF5, pOsAct1, pBdUBI10, pPvUBI1, pPvUBI2, pBdCCaMK, pSiCYCLOPS and pHvPht1.2 showed no *ccamk‐1* mutant complementation. Promoters pOsPGD1, pOsEIF5, pOsRS1, pOsRS2 and pOsRCc3 showed no *nfr1‐1* mutant complementation.

To further confirm the suitability of each promoter from our library for engineering symbiotic signalling in cereal roots, we investigated whether each promoter could function in a native symbiotic context and complement a legume mutant impaired for symbiosis. *Medicago truncatula* plants lacking a functional *CCaMK* gene are unable to form nodules (Levy *et al*., 2004; Mitra *et al*., 2004). Indeed, the *ccamk* mutant shows the strongest symbiotic phenotype of all symbiosis signalling pathway mutants (Kistner *et al*., 2005), making *CCaMK* an excellent gene to test for mutant complementation using our different promoters. We therefore generated constructs with each promoter from our genetic parts library driving *CCaMK*. We then transformed *ccamk* mutants with these constructs via *A. rhizogenes*‐mediated transformation and tested for restoration of nodulation (Figure 3a). This mutant complementation analysis revealed that most promoters from all three subgroups of our genetic parts library were able to successfully drive *CCaMK* expression and restore nodulation in the *ccamk* mutant background (Figure 3a). There were, however, a number of promoters which failed to drive *CCaMK* expression and restore nodulation, for example most notably the rice actin promoter (pOsAct1; McElroy *et al*., 1990), a commonly used constitutive promoter in cereal engineering (Figure 3a). Importantly, all the constitutive promoters that were unable to restore nodulation (pOsEIF5, pOsAct1, pBdUBI10, pPvUBI1 and pPvUBI2) also showed low or no activity in the *M. truncatula* LUC‐GUS assay (Figure 2). Statistical analysis reveals moderate correlation in constitutive promoter activities between the *M. truncatula* LUC‐GUS and *ccamk* mutant complementation assays, but no significant correlation between the *N. benthamiana* LUC‐GUS and the *ccamk* mutant complementation assays (Figure S1a). The correlation observed between both tests performed in *M. truncatula* roots is perhaps not surprising, but it is interesting to note that several of the constitutive promoters that performed best in the *ccamk* mutant complementation assays performed less well in the LUC‐GUS assays (Figure S1a). This suggests that high expression levels alone are not sufficient for restoring nodulation in the *ccamk* mutant background and that other factors may also be important, for example expression timing and cell/tissue‐specificity (Rival *et al*., 2012).

Most of the symbiosis‐related promoters from cereals could drive *CCaMK* expression and complement the *ccamk* mutant for nodulation (Figure 3a), suggesting that promoter function is retained between orthologous genes in cereal and model species. Almost all of the root‐specific promoters also functioned to restore nodulation in the *ccamk* mutant (Figure 3a). Analyses between promoter performance in the *M. truncatula ccamk* complementation experiments (Figure 3a) and the *N. benthamiana* LUC‐GUS assays (Figure 1) reveal good correlation for the symbiosis‐related promoters (Figure S1b), but no correlation for the root‐specific promoters (Figure S1c). This positive correlation for the symbiosis‐related promoters demonstrates that promoter analyses made in *N. benthamiana* leaves can be valid for *M. truncatula* roots. However, the lack of correlation for the root‐specific promoters between *N. benthamiana* leaves and *M. truncatula* roots suggests that results cannot always be applied between different species/tissues. Indeed, this is perhaps not that surprising given that the expression of root‐specific promoters is unlikely to be preserved between such different tissue types as leaves and roots. Overall, this analysis suggests that the *ccamk* mutant complementation offers a stringent screening strategy that takes into account more subtle effects than just expression level alone to test the functionality of promoters from our genetic parts library.

To further test the constitutive and root‐specific promoters in a symbiosis signalling context, we created constructs with different promoters driving expression of the Nod factor receptors *NFR1* and *NFR5* from *L. japonicus* (Madsen *et al*., 2003; Radutoiu *et al*., 2003). *Lotus japonicus nfr1* or *nfr5* mutant plants are unable to form nodules; therefore, we again used *A. rhizogenes‐*mediated transformation and restoration of nodulation to identify functional promoters. In our *nfr1* and *nfr5* mutant complementation experiments, we found that most of the tested promoters were successfully able to restore nodulation in both mutants (Figure 3b,c). However, some notable differences were observed between the different mutants; for example, pOsPGD1 and pOsEIF5 could be used to complement the *nfr5* mutant but not the *nfr1* mutant (Figure 3b,c). When comparing promoters between complementation of the *ccamk* mutant in *M. truncatula* (Figure 3a) and the *nfr1* and *nfr5* mutants in *L. japonicus* (Figure 3b,c), we also noticed some differences; for example, pBdUBI10 and pHvPht1.2 could not complement the *ccamk* mutant, but could complement the *nfr1* and *nfr5* mutants. No statistically significant correlations were observed between complementation of the *ccamk*,* nfr1* or *nfr5* mutants for either the constitutive or the root‐specific promoters (Figure S2). This suggests that the expression levels needed for *CCaMK*,* NFR1* and *NFR5* to function may be very different for each gene and that no straightforward rules can be defined for the ability of each individual promoter to allow complementation. We suggest that subtle differences in the expression pattern at the cell/tissue level and/or the timing of expression for each gene are important when attempting to engineer specific processes in plants and that these factors need to be further investigated for individual promoters in the future.

Combining our analyses in both *M. truncatula* and *L. japonicus*, we have categorized each promoter as having high, medium or low relative activity (Table 1; File S2). Three constitutive promoters complement all three symbiosis signalling pathway mutants to high levels and are therefore categorized as having high activity: pOsR1G1B, pOsCC1 and pBdEF1α (Table 1; File S2). The constitutive pAtUBI10 promoter, which was discussed earlier because it showed high activity levels in the LUC‐GUS assays in *N. benthamiana* (Figure 1) and *M. truncatula* (Figure 2), continued to show good activity in the nodulation tests and therefore also received a high activity rating (Table 1; File S2). A number of other promoters also complement all three legume mutants, albeit to lower levels, and these have been categorized as having medium activity: the constitutive promoters pZmPIP2.1 and pZmEF1α, and the root‐specific promoters pIbSRD1, pSlREO, pZmRsyn7 and pHvPht1.1. We particularly recommend these high and medium activity promoters for engineering symbiosis signalling. By comparing across the tests in *N. benthamiana* leaves and legume roots, we have identified a number of promoters with high activity levels in both systems and we would specifically recommend these promoters for engineering both the leaves and roots of dicotyledonous plants: pBdEF1α, p35S, pAtUBI10, pOsUBI3, pOsR1G1B, pMtCCaMK, pLjCCaMK, pOsCCaMK, pSiCCaMK and pMtPT2.

### Characterization of promoter expression in barley

**Figure 4:**
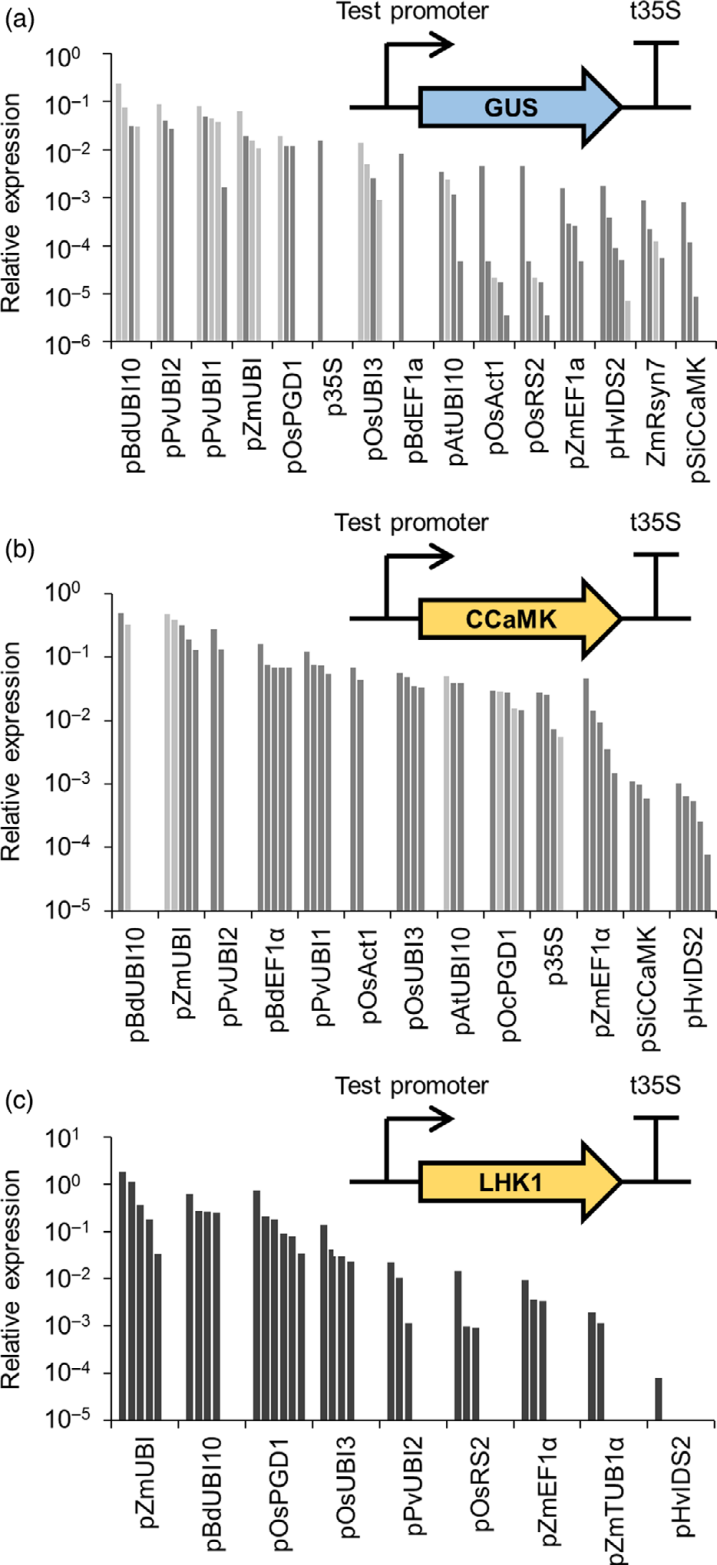
Different promoters from the standard genetic parts library give varying levels of transgene expression levels in barley. Real‐time quantitative reverse transcription PCR analysis of transgene expression in roots from T0 barley plants transformed with constructs containing promoters from the standard genetic parts library driving expression of *GUS* (a), *CCaMK* (b) or *LHK1* (c) (see figure insets). Each bar represents an individual plant line. In panels (a) and (b), single and two‐copy lines are shown in dark and light grey, respectively. Copy number analysis was not performed for lines in panel (c).

Our initial tests revealed that promoters from our genetic parts library are functional in dicotyledonous plants (*N. benthamiana, M. truncatula* and *L. japonicus*), but since our aims are to engineer cereal roots we considered it essential to confirm and empirically test the functionality of these promoters in a cereal species. We selected barley (*Hordeum vulgare* cv. Golden Promise) as our target cereal species because it has an established high‐efficiency transformation system (Bartlett *et al*., 2008). We tested whether the promoters from our genetic parts library functioned in a cereal by transforming barley with all 46 promoter‐GUS constructs. Analysis of roots from T0 transgenic barley plants (on average five plants per construct) revealed that only 14 promoters gave positive GUS staining results (Table S1 and Figure S3); most of these were categorized as constitutive promoters, although one symbiosis‐related promoter (pSiCCaMK) and two root‐specific promoters (pHvIDS2 and pOsRS2) showed positive GUS staining in some transgenic plants (Table S1). Visual analysis of these T0 barley roots suggested different levels of GUS activity; for example, pAtUBI10, pPvUBI1, pZmUBI, pBdEF1α and pOsUBI3 gave stronger GUS staining than other promoters (Figure S3). This simple GUS staining analysis allowed us to identify those promoters that were functional in barley, but to characterize these promoters in greater detail we also performed real‐time quantitative reverse transcription PCR (qRT‐PCR) to quantify the amount of *GUS* transcript and thereby determine the relative activity of each promoter. This qRT‐PCR analysis revealed that almost all the constitutive promoters showed stronger *GUS* expression than the root‐specific or symbiosis‐related promoters (Figure 4a and File S2). Importantly, we identified several promoters which showed expression levels higher than or comparable with the common promoters traditionally used in cereal engineering (pZmUBI, p35S and pOsAct1), namely pBdUBI10, pPvUBI2, pPvUBI1, pOsPGD1, pOsUBI3, pBdEF1α and pAtUBI10. These promoters have previously been individually described in the literature, but due to lack of side‐by‐side comparisons between different promoters, it was not possible to infer the relative strength and suitability of these promoters.

To confirm the relative activity of the subset of promoters expressed in barley, we generated transgenic barley plants expressing codon‐optimized versions of *M. truncatula CCaMK* and the *L. japonicus* symbiosis signalling gene *LHK1* (Tirichine *et al*., 2007) under the control of each different promoter. Root material was harvested from T0 barley plants, and qRT‐PCR was performed to determine the relative expression levels of *CCaMK* (Figure 4b) and *LHK1* (Figure 4c). This analysis confirmed that the constitutive promoters showed higher levels of expression than the root‐specific or symbiosis‐related promoters (Figure 4). The pBdUBI10 and pZmUBI promoters consistently showed the strongest levels of expression. Strong correlation in relative promoter activity was observed between the results obtained for the *GUS*,* CCaMK* and *LHK1* transgenes (Figure S4), demonstrating that the transgene used made little difference to the relative expression levels obtained with a specific promoter. Our analysis confirms previous observations in cereals that transgene expression driven by the 35S promoter (p35S) is lower than the maize ubiquitin promoter (pZmUBI1; Lee *et al*., 2007), but we have also identified a subset of promoters which give equal or stronger expression in cereals than the 35S promoter (Figure 4 and File S2). In addition, we have identified constitutive promoters which give relatively equal levels of expression in barley (e.g. pAtUBI10 and pOsUBI3, and pPvUBI1 and BdEF1α; Figure 4b), and we recommend these promoters for engineering multigenic traits in cereals where it is important to achieve equivalent expression levels of each transgene. Based on this analysis, we have classified the promoters as displaying either high, medium or low activity in cereal roots (Table 1; File S2). Promoters which give high activity in cereals include pBdEF1α, pZmUBI, pBdUBI10, pPvUBI1 and pPvUBI2 (Table 1), and we recommend these for achieving high levels of transgene expression in cereals.

### Assessing the importance of terminators for transgene expression in *Medicago truncatula* and barley

**Figure 5:**
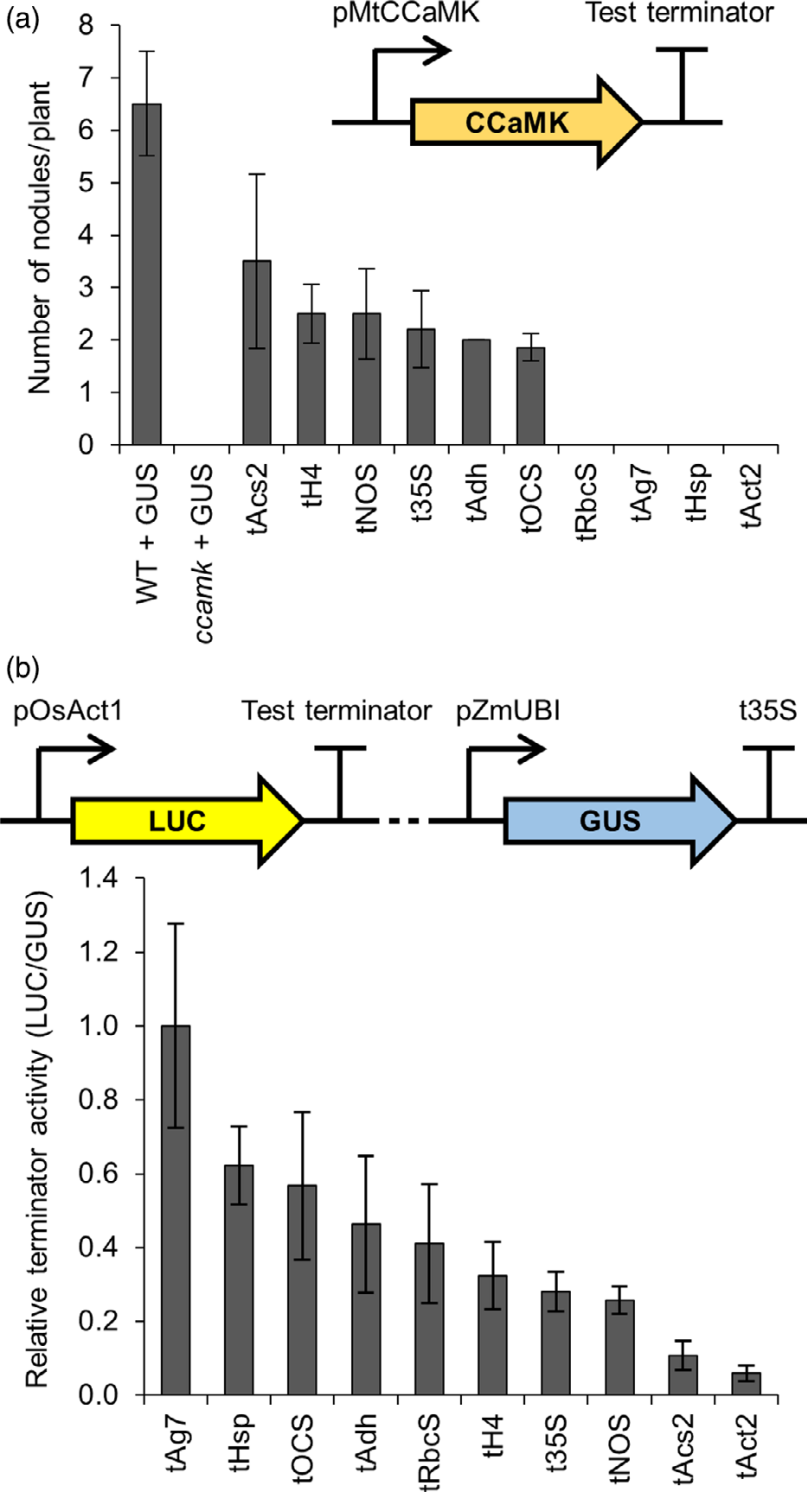
Terminators from the standard genetic parts library show varying levels of functionality in different plant species. (a) Constructs containing terminators from the standard genetic parts library terminating expression of the *CCaMK* gene (inset) were transformed into *Medicago truncatula ccamk‐1* mutant plants via *Agrobacterium rhizogenes*‐mediated transformation. The numbers of root nodules on these plants were counted three weeks after inoculation with *Sinorhizobium meliloti*. Data represent mean ± standard error. An average of 9 independently transformed *M. truncatula* plants were assessed per construct (dsRED in the T‐DNA confirmed transformation). Control wild‐type (WT) and *ccamk* mutant plants were transformed with a pAtUBI10‐GUS construct (GUS). (b) Multigene constructs containing terminators from the standard genetic parts library driving expression of the firefly luciferase (LUC) reporter gene, plus a constitutively expressed β‐glucuronidase gene (GUS; see figure inset), were expressed in barley (T0 roots). GUS and LUC reporter gene activities were quantified using a plate reader, and terminator activity was calculated by determining the LUC to GUS ratio (with normalization to tAg7 terminator). Data represent mean ± standard error. An average of 5 T0 barley root samples with transgene copy numbers varying between 1 and 4 were assessed per construct. Dotted line in figure inset represents 3.6 kb of plasmid sequence that was unchanged between constructs containing different test terminators. Terminators tRbcS, tAg7, tHsp and tAct2 showed no *ccamk‐1* mutant complementation.

Terminator sequences can influence the level of transgene expression in plants (Ingelbrecht *et al*., 1989; Nagaya *et al*., 2010); therefore, in addition to characterizing promoters from our library of genetic parts, we also defined and tested 10 terminator sequences (see File S1) for their mRNA‐stabilizing capacity. We created constructs with different terminator sequences, but with an identical promoter (pMtCCaMK for complementation of the *M. truncatula ccamk* mutant and pOsAct1 for tests in barley). Six of the ten terminator sequences allowed complementation of the *ccamk* mutant, leading to the formation of nodules, while four of the terminator constructs showed no complementation (Figure 5a; dsRED in the T‐DNA confirmed transformation). For tests in barley, we monitored the expression of a luciferase (*LUC*) reporter gene with each test terminator, using a constitutively expressed *GUS* as a transformation control (Figure 5b). All 10 terminators were functional in barley in this assay, although there was a gradation of LUC activity dependent on the different terminators (Figure 5b). There was no correlation between the terminators optimal for *ccamk* complementation in *M. truncatula* and those that gave the highest *LUC* expression in barley (Figure S5). This may be explained by different relationships between the terminators and the two promoters (pCCaMK and pOsAct1) used and/or transgene of interest being expressed (*CCaMK* and *LUC*), or it may reflect species differences in the behaviour of the terminators. These results point at the importance of selecting the right promoter/terminator combination for optimal transgene expression.

### Codon optimization and intron‐mediated enhancement increase transgene expression in barley

**Figure 6:**
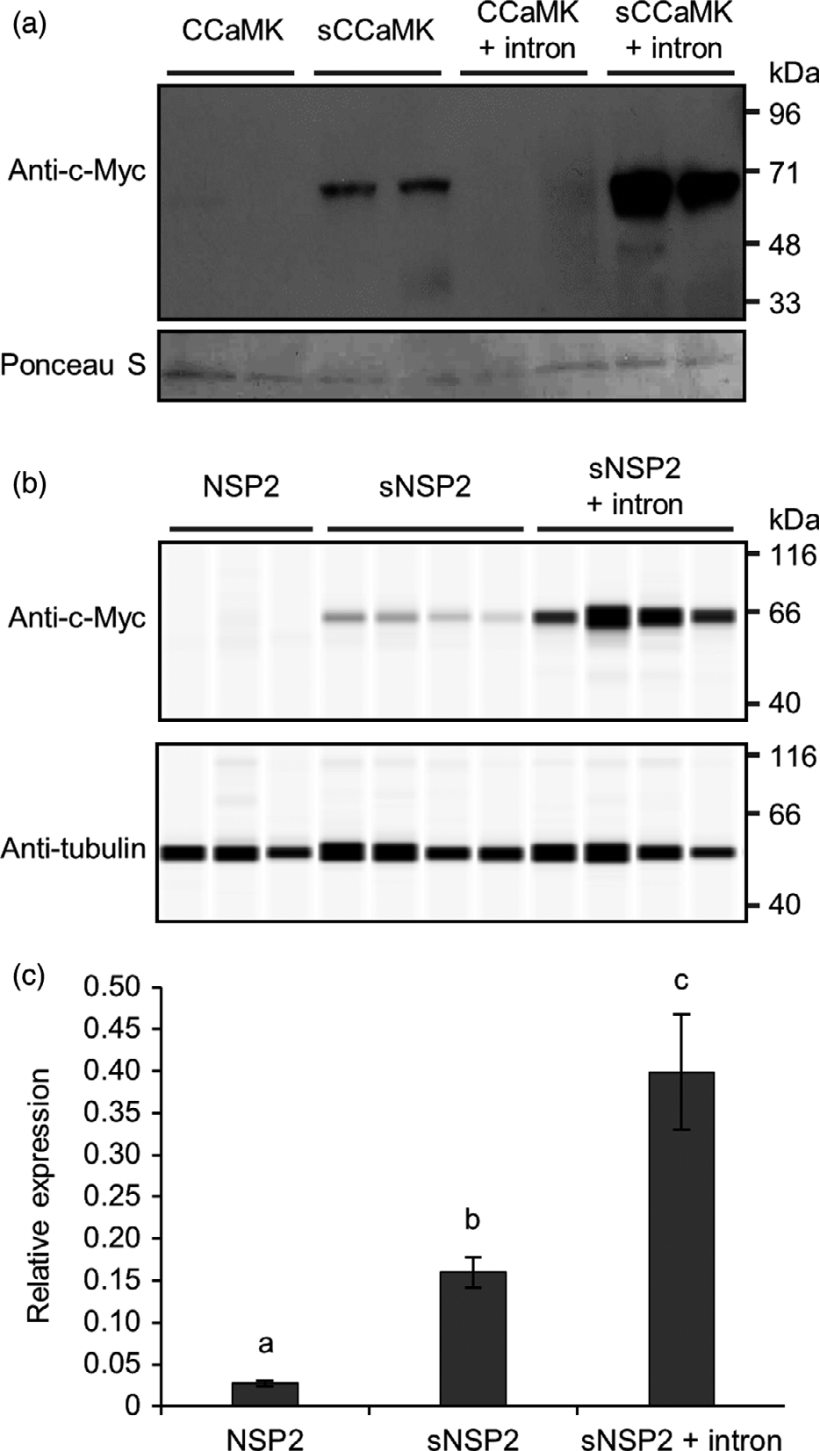
Codon optimization and intron‐mediated enhancement increase transgene expression in barley. (a) Total protein was extracted from T1 barley plants transformed with constructs over‐expressing different versions of *Medicago truncatula* CCaMK tagged with an N‐terminal 3xMyc tag. Western blotting was performed using anti‐c‐Myc antibody and chemiluminescence to detect 3xMyc‐CCaMK protein (expected molecular weight = 66 kDa; upper panel); protein loading control (Ponceau S; lower panel). (b) Total protein was extracted from T0 barley plants transformed with constructs over‐expressing *M. truncatula* NSP2 tagged with a C‐terminal 3xMyc‐tag. NSP2‐3xMyc was detected via a WES Protein Simple system using anti‐c‐Myc antibody as indicated (expected molecular weight = 60 kDa); protein loading control using anti‐α‐tubulin antibody. (c) Real‐time quantitative reverse transcription PCR analysis of T0 barley plants from (b). Data represent mean ± standard error of results from six independent transgenic plant lines. Codon‐optimized sequences of transgenes are denoted by lower case ‘s’; the synthetic *Arabidopsis thaliana UBI10* intron was additionally included in sequences as indicated (see File S1 for sequence information). *CCaMK* and *NSP2* were expressed under the control of the maize ubiquitin promoter (pZmUBI); each lane in (a) and (b) contains protein extracted from an independent transgenic line.

Codon optimization and intron‐mediated enhancement can be individually used to increase transgene expression in plants (see recent reviews by Laxa, 2016; Webster *et al*., 2017); however, these approaches have not been combined together or tested side‐by‐side using the same transgene of interest. We therefore felt it important to compare the two approaches and determine whether they can be used in conjunction to maximally increase transgene expression in a cereal. We tested the relative importance of both codon optimization and intron‐mediated enhancement for expression of two symbiotic genes in barley: *CCaMK* (Gleason *et al*., 2006; Tirichine *et al*., 2006) and *NSP2* (Kalo *et al*., 2005; Smit *et al*., 2005). In all cases, we used the maize ubiquitin promoter (pZmUBI), a 3xMyc tag and the 35S terminator (t35S). We compared expression of the native *M. truncatula* cDNA sequence, a cereal codon optimized version of the cDNA (see Experimental Procedures) and of cDNA sequences containing a synthetic intron taken from the *Arabidopsis thaliana UBI10* gene (insertion of this intron has been shown to increase expression levels; Bartlett *et al*., 2009). These constructs were transformed into barley, and root material from independent transgenic lines was analysed by Western blotting to detect protein levels (Figure 6). For both CCaMK and NSP2, no protein expression was detected from the genes lacking codon optimization. With codon optimization alone, a low level of expression was observed, but only when codon optimization was combined with the addition of the synthetic intron were high levels of protein expression observed for both CCaMK and NSP2 (Figure 6a,b). The levels of NSP2 protein correlated directly with mRNA levels (Figure 6c). Indeed, the codon‐optimized version of *NSP2* showed approximately sixfold higher expression than the native cDNA version, and this was increased further to 15‐fold higher expression with the codon‐optimized version of *NSP2* that included the synthetic intron (Figure 6c). It is interesting to note that codon optimization alone increased the steady‐state mRNA levels of *NSP2* transcript (Figure 6c), suggesting that transcription and/or mRNA stability contributed to the observed changes in protein level. Overall, we recommend the combined use of codon optimization and inclusion of a synthetic intron to achieve maximal transgene expression and protein levels in cereal engineering.

## Conclusions

Our work has characterized 46 promoters in different plant species, and we recommend these promoters for general use within the research community. We have classified the promoters based on the levels of activity in different plant species and tissue types (Table 1 and Figure S6) and would encourage potential users to carefully select promoters based on the experimental conditions which best match their needs. We have identified seven promoters which are especially useful for plant synthetic biology since they drive high levels of transgene expression in leaves and roots of both dicotyledonous and monocotyledonous plants: pBdEF1α, p35S, pAtUBI10, pOsUBI3, pZmUBI, pBdUBI10 and pOsPGD1 (category 1, Table 1). In addition to these promoters, we would also specifically recommend pPvUBI2, pPvUBI1, pOsAct1 and pOsRS2 as a suite of promoters suitable for regulating gene expression in cereal roots (categories 3 and 4, Table 1). We advocate using the promoters in category 2 (Table 1) as those which are best for leaf and root expression in dicotyledonous plants, and the sets of promoters in categories 5 and 6 as those which show preference for expression in either roots or leaves.

Our findings reveal that appropriate selection of terminator sequence is an important factor for transgene expression in both dicotyledonous and monocotyledonous plants. We specifically recommend using the t35S, tNOS, tH4, tAdh and tOCS terminators as these are functional in both dicotyledonous and monocotyledonous plant species.

We strongly recommend the use of codon optimization to increase transgene expression levels in cereals, but we also advise the additional inclusion of synthetic introns to further increase transgene expression levels.

## Experimental procedures

### Cloning of constructs for plant transformation

All promoter and terminator sequences included in our library of standard genetic parts were cloned into plasmids for further subcloning into multigene plant expression vectors using Golden Gate cloning methods (Patron *et al*., 2015; Weber *et al*., 2011). Since Golden Gate assembly relies on type IIS restriction enzymes, we first ‘domesticated’ all promoters and terminators by removing any internal restriction enzyme sites (*Bsa*I, *Bpi*I, *Esp*3I and *Dra*III) which would interfere with downstream cloning. All ‘domesticated’ sequences were synthesized by commercial DNA synthesis and provided as sequence‐verified clones in Level 0 Golden Gate vectors (spectinomycin resistance; 100 mg/L spectinomycin used for selection). All promoters were cloned into a Level 0 Golden Gate vector flanked by GGAG and AATG fusion sites, and all terminators were cloned into a Level 0 Golden Gate vector flanked by GCTT and CGCT fusion sites, according to the common syntax described by Patron *et al*. (2015). DNA synthesis was also employed to create constructs containing ‘domesticated’ coding sequences, including codon‐optimized genes and genes with addition of a synthetic intron. Codon optimization was performed using the Invitrogen GeneArt gene synthesis online tool based on codon usage frequency tables for *Zea mays*. The coding sequences of the *CCaMK*,* NSP1* and *NSP2* genes had a synthetic intron taken from the *Arabidopsis thaliana UBI10* gene added 200–500 bp after the start codon (Bartlett *et al*., 2009).

Single gene constructs (e.g. containing promoter‐gene‐terminator) were cloned into Level 1 Golden Gate vectors (ampicillin resistance; 100 mg/L ampicillin used for selection) via a standard Golden Gate reaction using 100 ng of each Golden Gate vector, *Bsa*I and T4 DNA ligase, as described by Weber *et al*. (2011). Correct Level 1 construction was confirmed by colony PCR. Colony PCR products were performed in 10 μL final volume, using 5 μL GoTaq^®^ Green Master mix (Promega, Madison, WI), 1 μL of each plasmid and/or gene‐specific primer (20 μm; Table S2), 3 μL dH_2_O and a small amount of bacterial colony as a template. PCR amplification was performed with an initial denaturation step of 95 °C for 10 min; followed by 30 cycles of 95 °C for 10 s, 55 °C for 20 s, 72 °C for 1 min/kb; and a final elongation step at 72 °C for 5 min. Positive clones were cultured overnight in LB broth containing 100 mg/L ampicillin, and plasmid DNA was isolated using QIAprep^®^ Spin Miniprep kit (Qiagen, Hilden, Germany) according to the manufacturer's instructions. Sanger sequencing (Eurofins Mix2Seq kit) with plasmid or gene‐specific primers was performed according to the manufacturer's instructions to verify the cloned insert. Sequences were aligned using Vector NTI (Life Technologies, Carlsbad, CA) and Clustal Omega.

Multigene constructs were cloned into Level 2 Golden Gate vectors (kanamycin resistance; 25 μg/mL used for selection) via standard Golden Gate reaction using 100 ng of each Golden Gate vector, T4 DNA ligase and either *Bpi*I or *Bpi*I and *Bsa*I, as described by Weber *et al*. (2011). All Level 2 constructs were confirmed by colony PCR with plasmid and/or gene‐specific primers (as above), restriction enzyme digestion using common commercial enzymes yielding fragments with unique digestion patterns (e.g. *Hind*III, *Bam*HI, *Eco*RI), and complete resequencing by commercial next‐generation sequencing (IMGM Laboratories, Martinsried, Germany). A summary of all Levels 0, 1 and 2 constructs used in this study is provided in File S3. Sequence‐verified Level 2 constructs were transformed into *A. tumefaciens* strain GV3101 (for transformation of *N. benthamiana*), *A. tumefaciens* strain AGL1 (for transformation of barley) and *A. rhizogenes* strain AR1193 (for transformation of *M. truncatula*), and verified by colony PCR.

### Transformation of *Nicotiana benthamiana*

*Agrobacterium tumefaciens* strain GV3101 carrying the appropriate sequence‐verified Level 2 construct was grown in overnight culture at 28 °C in LB broth containing 50 mg/L rifampicin, 40 mg/L gentamycin and 25 mg/L kanamycin. The culture was centrifuged for 15 min at room temperature (2900 ***g***), and the pellet was resuspended in infiltration buffer (10 mm MES; 10 mm MgCl_2_; 140 μm acetosyringone). The final OD_600_ was adjusted to 0.3–0.4 and bacteria incubated in the dark for 2 h at room temperature. Using a syringe, three leaves of 3‐ to 4‐week‐old tobacco plants were infiltrated per construct. After 60–72 h, three 1‐cm‐diameter leaf discs were punched from each plant and used for further characterization.

### Transformation of *Medicago truncatula* and *Lotus japonicus* and nodulation assays

Seeds of *M. truncatula* cv Jemalong A17 and *ccamk‐1* (*dmi3‐1*; TRV25) were scarified with sandpaper or 98% H_2_SO_4_, surface‐sterilized in 10% sodium hypochlorite solution, imbibed in sterile water and plated on 1% deionized water agar. After stratification at 4 °C for a minimum of four days, seeds were germinated overnight at room temperature. Seedlings were transformed with *A. rhizogenes* strain AR1193 carrying the appropriate sequence‐verified Level 2 construct as described by Boisson‐Dernier *et al*. (2001). Three weeks after transformation, plants were visually screened using a stereoscope with UV lamp and only those plants with positive dsRED fluorescence were selected for nodulation. Plants for nodulation were transferred to a 1:1 mix of sand:terragreen (Oil‐Dri Company, Wisbech, UK) and inoculated with *Sinorhizobium meliloti* strain 1021 (OD_600_ = 0.03). Nodulation was scored four weeks after inoculation.

*Lotus japonicus* hairy roots were induced by infection of 6‐day‐old seedlings growing on vertical 0.8% Phytagel (Sigma) plates with half‐strength B5 salts and vitamins as described (Hansen *et al*., 1989). Three weeks after infection, primary roots were removed, and the chimeric plants transferred to plastic boxes containing 1:4 leca:vermiculite mix. Plants were inoculated with *Mesorhizobium loti* strain R7A and screened for nodulation five weeks after inoculation.

### Transformation of barley and copy number analysis

Barley (*Hordeum vulgare* cv. Golden Promise) was transformed as described by Bartlett *et al*. (2008). Leaf tissue (1–2 cm leaf material) from individual hygromycin‐resistant transgenic barley plants was frozen in liquid nitrogen, and copy number analysis was performed on this material using a TaqMan assay by IDna Genetics (Norwich, UK).

### GUS staining and quantification of GUS and luciferase activities

Transgenic plant material (excised barley roots) was submerged in GUS staining buffer: 100 mm NaPO_4_ buffer, pH 7; 10 mm EDTA; 0.1% Triton X‐100; 1 mm ferrocyanide; 2 mm 5‐bromo‐4‐chloro‐3‐indolyl‐β‐d‐glucuronic acid (X‐GlcA). Samples were incubated at 37 °C overnight (18–20 h), and the staining reaction was stopped by replacing the GUS staining buffer with 70% ethanol. GUS‐stained tissues were observed using a Leica DM 6000 microscope with DFC420 colour camera (Leica, Wetzlar, Germany).

Luciferase activity was measured using the Luciferase Assay System (Promega, E1500). Frozen tissue powder (from three 1‐cm‐diameter *N. benthamiana* leaf discs, or 50–100 mg of *M. truncatula* or barley root material) was homogenized in 250 μL of 1 × Cell Culture Lysis Reagent containing protease inhibitor (cOmplete™ Mini Protease Inhibitor Cocktail, Roche, Basel, Switzerland). After 10‐min centrifugation at 20 000 ***g*** in a microcentrifuge (4 °C), the supernatant was transferred into a new tube. For the luciferase measurement, 20 μL of each supernatant was pipetted into white flat‐bottom 96‐well microplates (Greiner Bio‐One) and luminescence was measured using a Varioskan^®^ Flash multimode plate reader (Thermo Scientific, Waltham, MA). During the measurement, 100 μL Luciferase Assay Reagent was automatically injected into each well and luminescence was recorded 20 times for 1000 ms each. Only the last value (collected about 20 s after injection) was then used for the calculations. For the measurement of GUS activity, 40 μL of the supernatant was mixed with 100 μL of warm (37 °C) MUG assay buffer: 50 mm phosphate buffer, pH 7; 10 mm EDTA; 0.1% Triton X‐100; 0.1% sodium lauryl sarcosine; 10 mm β‐mercaptoethanol; 2 mm 4‐methylumbelliferyl‐β‐d‐glucuronide hydrate (MUG). To stop the reaction, three aliquots of 20 μL were immediately transferred into black flat‐bottom 96‐well microplates (Greiner Bio‐One) containing 180 μL of 200 mm Na_2_CO_3_. The remainder of the reaction was incubated at 37 °C for another 60 min, after which another three aliquots of 20 μL were mixed with Na_2_CO_3_ solution. MUG fluorescence from both time points (t0 and t60) was measured for 100 ms using the Varioskan^®^ Flash multimode plate reader (Thermo Scientific) using excitation and emission wavelengths of 365 and 450 nm, respectively. To determine promoter activity, the GUS/luciferase activity ratio for each sample was first calculated by dividing the average photon counts (t60 − t0) from all three GUS measurements by the photon counts from the luciferase measurement. For the graphs, the average GUS/luciferase ratio of all biological replicates for each promoter was divided by that of the pOsAct1 promoter. Terminator activity was determined in the same way, but by using the luciferase/GUS ratios.

### Real‐time quantitative reverse transcription PCR (qRT‐PCR)

Root tissue of three‐month‐old hygromycin‐resistant barley T0 plants was flash‐frozen in liquid nitrogen and stored at −80 °C. Frozen material was ground into a fine powder in 2‐mL microcentrifuge tubes containing a 4‐mm‐diameter stainless steel ball (Bearing Supplies) using a mixer mill (Retsch) by shaking for 1.5 min at 23 strokes per second. About 50 mg of frozen tissue powder was used for RNA extraction, using the RNeasy^®^ Plant Mini Kit (Qiagen) and following the manufacturer's instructions. RNA was eluted from the RNeasy spin column using 30 μL of RNase‐free dH_2_O, and the quality of the eluted RNA was assessed by measuring the absorbance at 260 and 280 nm using the NanoDrop ND‐1000 spectrophotometer (NanoDrop Technologies, Wilmington, DE). In addition, about 1 μg of each RNA sample was analysed by agarose gel electrophoresis to confirm the presence of rRNA double bands. RNA samples were then treated with RQ1‐DNase (Promega) in a final volume of 20 μL, using 500 ng of RNA and following the manufacturer's instructions. Half of the DNA‐free RNA sample was used for cDNA synthesis; the other half was kept as negative control for subsequent qRT‐PCR mixtures to confirm the absence of genomic DNA. For reverse transcription, the SuperScript^®^ III Reverse Transcriptase kit (Life Technologies) and Oligo d(T)12–18 primer (Invitrogen, Carlsbad, CA) were used, according to the manufacturer's instructions in a total volume of 20 μL per sample. The cDNA was diluted 1:4 in RNase‐free water. qRT‐PCR analysis was carried out in triplicates in a final volume of 10 μL containing 2 μL of each primer (2.5 μm) and 6 μL of a master mix with 5 μL SYBR^®^ Green JumpStart™ Taq ReadyMix™ (Sigma, St. Louis, MO) and 1 μL of cDNA. Reactions were performed on a Bio‐Rad CFX96™ with following cycling parameters: 94 °C for 2 min, followed by 40 cycles of 94 °C for 15 s, 60 °C for 1 min, read fluorescence. qRT‐PCR primers were designed to amplify a product of about 90 bp at the 3′ end of the transcript using Primer3web version 4.1.0 (http://primer3.ut.ee/) and are listed in Table S2. Primer efficiencies were analysed using a dilution series of pooled cDNA as qRT‐PCR templates. Primer pairs were selected if average Ct values of the dilution series followed a linear standard curve (*R* ^2^ > 0.96) and if they had an efficiency of about 90%–110%. It was also verified that primers do not amplify control samples without cDNA template or on DNase‐treated RNA. For each cDNA sample, the average Ct value of the *GAPDH* reference gene (Bartlett *et al*., 2009) was subtracted from the average Ct value of the *GUS*,* sCCaMK*,* NSP2* or *sNSP2* genes. This difference was then linearized with the formula 2 to the power of ‐[Ct difference]. The averages of all biological replicates were then presented as ‘relative expression’ for each construct. For *LHK1* analysis, experiments were performed as described above with the following exceptions: reactions were performed on a Roche LightCycler^®^ 480, and expression relative to the housekeeping genes was calculated using per amplicon PCR efficiency calculations from LinRegPCR (Ramakers *et al*., 2003).

### Western blotting

For the selection of barley transformants, T1 seeds were germinated on 0.7% agar (Formedium) containing 100 mg/L hygromycin (Roche). Plates were incubated at 4 °C in the dark for 3–4 days and then moved to room temperature and left wrapped in foil for 2 days. On the third day, the foil was removed and the plates were left at room temperature and lighting for an additional 2–3 days. About 1 cm of root material of plants surviving the hygromycin‐selection was collected and frozen for DNA extraction and hygromycin copy number analysis. The remainder of the root tissue was collected, flash‐frozen, stored at −80 °C and ground into a fine powder in 2‐mL microcentrifuge tubes containing a 4‐mm‐diameter stainless steel ball (Bearing Supplies) using a mixer mill (Retsch) by shaking for 1.5 min at 23 strokes per second under constant cooling. The frozen powder was homogenized in 200 μL ice‐cold extraction buffer (50 mm Tris‐HCl pH 7.5; 400 mm NaCl; 1 mm EDTA; cOmplete™ Mini Protease Inhibitor Cocktail, Roche) per 100 mg of sample. Samples were left on ice for 20 min then spun in a centrifuge at 20 000 ***g*** and 4 °C for 10 min. The supernatant was transferred into a new tube and mixed 1:1 with 2 × Laemmli buffer (Bio‐Rad, Hercules, CA) before boiling at 95 °C for 10 min. 20 μL of each sample was loaded undiluted on 10% precast SDS gels (Bio‐Rad). Transfer of proteins to PVDF membrane (Thermo Scientific) was carried out using the Trans‐Blot SD transfer apparatus (Bio‐Rad) at 4 °C for 2 h at 100 V. The membrane was washed three times in TBS‐T (100 mm Tris, pH 7.5; 150 mm NaCl; 0.1% (v/v) Tween 20) and incubated for one hour on a rocking platform at room temperature in blocking solution (TBS‐T containing 5% (w/v) skimmed milk powder). The blots were incubated at 4 °C overnight with anti‐c‐Myc mouse monoclonal primary antibody (Thermo Fisher, clone 9e10) at a 1:1000 dilution in blocking buffer. The blots were washed six times in TBS‐T and then probed with secondary antibody (anti‐mouse antibody conjugated with horse reddish peroxidase (HRP; Sigma) at a dilution of 1:2000 in blocking buffer for 2 h at room temperature and washed three times in TBS‐T. Detection of chemiluminescence was carried out using ECL Prime (GE Life Sciences, Marlborough, MA) for 3 min. Staining with Ponceau S (Sigma) was performed following the manufacturer's instructions to control for loading. Preparation of barley T1 root material for analysis on the WES system (Protein Simple) was performed as described above, with the exception that RIPA lysis buffer (Protein Simple; 040‐483) was used as extraction buffer at a tissue to buffer ratio of 1:2 (w/v). For the WES analysis, the anti‐c‐Myc mouse monoclonal primary antibody (ThermoFisher, clone 9e10) and monoclonal anti‐α‐tubulin primary antibody (Sigma, clone B‐5‐1‐2) were used at a 1:50 dilution, as recommended by the manufacturer's instructions, and samples were diluted 1:10 in WES sample buffer (Protein Simple, PS‐MK15). WES analysis was carried out on the WES system using the Wes‐Mouse (12–230 kDa) Master Kit with Split Buffer (Protein Simple, PS‐MK15) according to the manufacturer's instructions. WES data were analysed using Compass software (http://www.proteinsimple.com/compass/downloads/).

## Conflict of interest

The authors declare no conflict of interest.

## Author contributions

D.F., E.M., D.R., C.R., S.R., J.S., W.A.H., G.E.D.O and J.B.M. designed the research. D.F., A.V.K., E.S., E.M., D.R., A.B. and J.B.M. performed the research. J.B.M. wrote the article with input from D.F. and G.E.D.O.

## Supporting information


**Figure S1** Rank correlation analysis between promoter activities tested by LUC‐GUS assays in *Nicotiana benthamiana* and *Medicago truncatula*, and *ccamk* mutant complementation in *M. truncatula*.

**Figure S2** Rank correlation analysis between promoter activities tested by *ccamk* mutant complementation in *Medicago truncatula*, and *nfr1* and *nfr5* mutant complementation in *Lotus japonicus*.

**Figure S3** GUS staining reveals that promoters from the standard genetic parts library show different levels of activity in barley.

**Figure S4** Rank correlation analysis between promoter activities tested by *GUS*,* CCaMK* and *LHK1* expression in barley.

**Figure S5** Rank correlation analysis between terminator activities tested by *ccamk* mutant complementation in *Medicago truncatula* and *LUC* expression in barley.

**Figure S6** Heat‐map summary of all promoter testing data.

**Table S1** Summary of GUS staining results in barley.
**Table S2** Primers used in this study.

**File S1** References and sequences of promoters, terminators and coding sequences used in this study.

**File S2** Summary of all genetic part characterisation data presented in this study.

**File S3** Summary of all Golden Gate constructs used in this study.
