# Supplementary figures and images for: Characterizing standard genetic parts and establishing common principles for engineering legume and cereal roots
Source: Plant Biotechnol J. 2019 May 23;17(12):2234–45. doi: 10.1111/pbi.13135 (PMC6835126; doi:10.1111/pbi.13135)

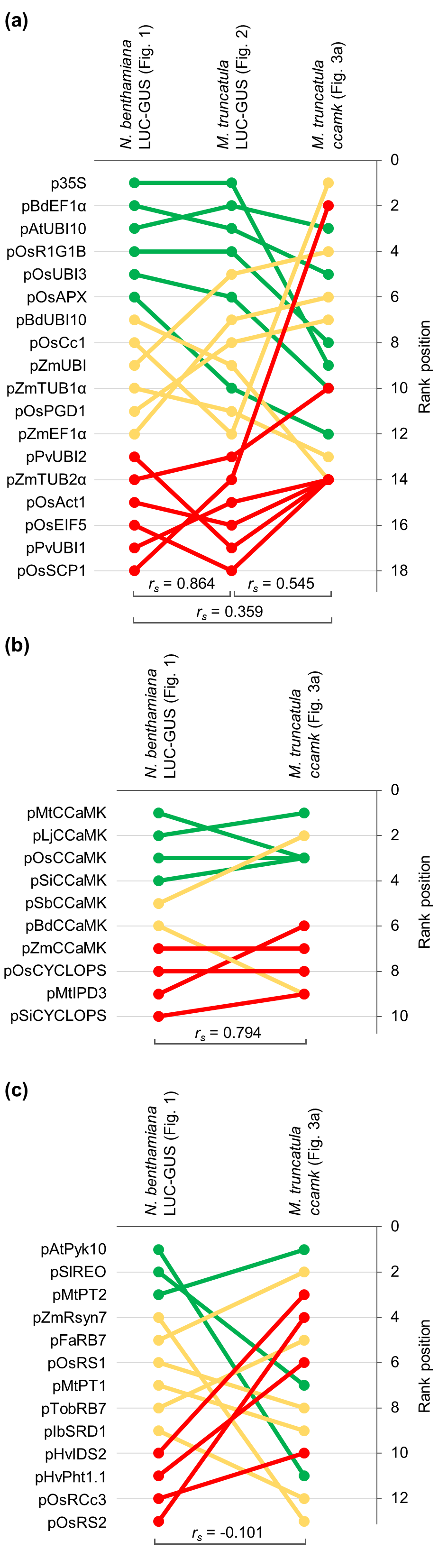

Supplement: Supplementary file 1 — Figure S1 Rank correlation analysis between promoter activities tested by LUC‐GUS assays in Nicotiana benthamiana and Medicago truncatula, and ccamk mutant complementation in M. truncatula. [file PBI-17-2234-s007.tif]

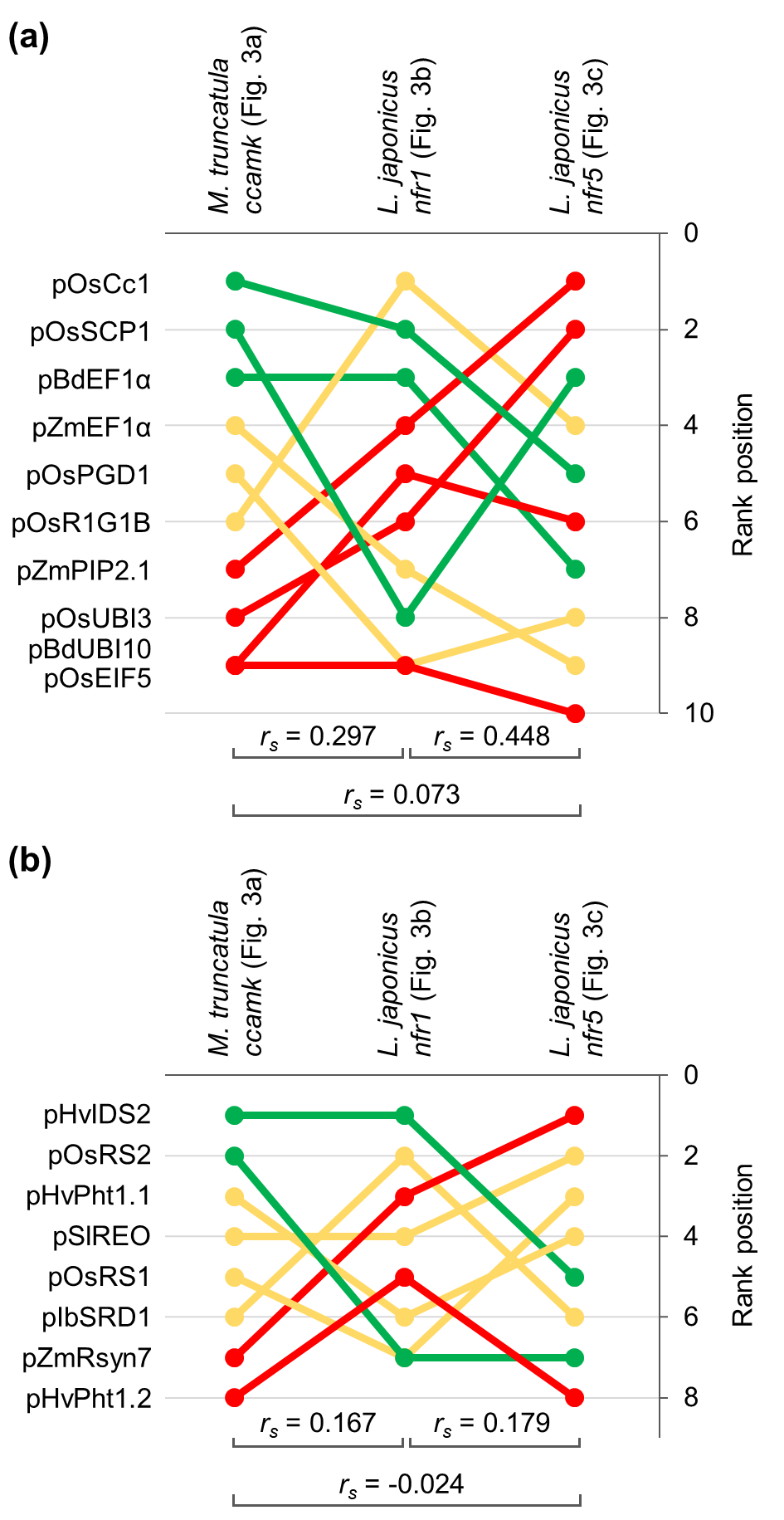

Supplement: Supplementary file 2 — Figure S2 Rank correlation analysis between promoter activities tested by ccamk mutant complementation in Medicago truncatula, and nfr1 and nfr5 mutant complementation in Lotus japonicus. [file PBI-17-2234-s006.tif]

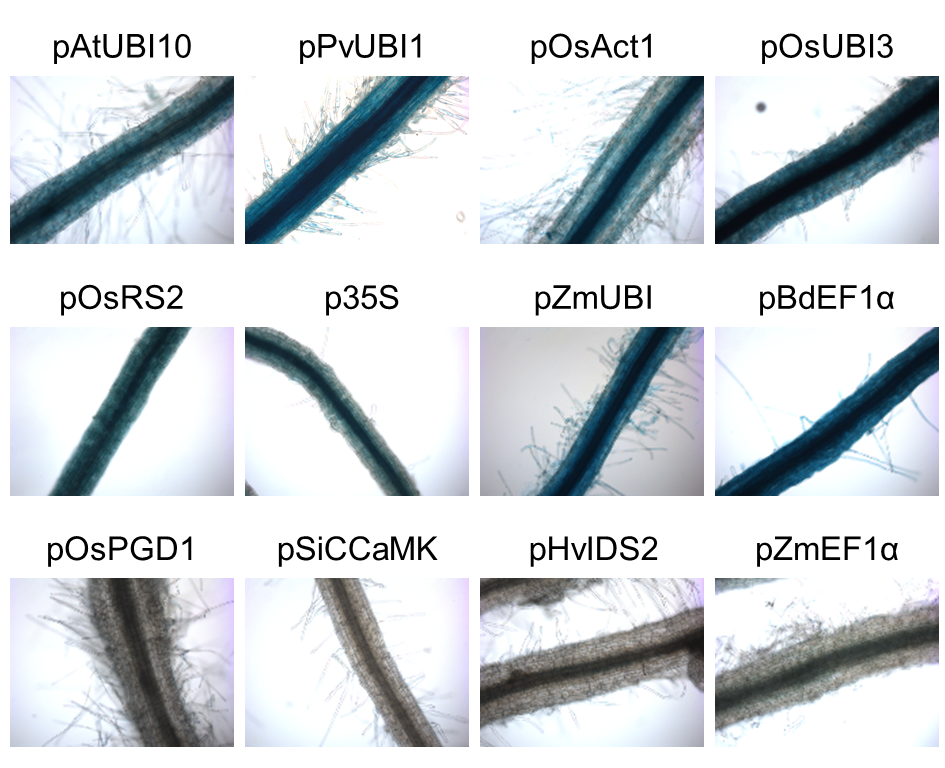

Supplement: Supplementary file 3 — Figure S3 GUS staining reveals that promoters from the standard genetic parts library show different levels of activity in barley. [file PBI-17-2234-s005.tif]

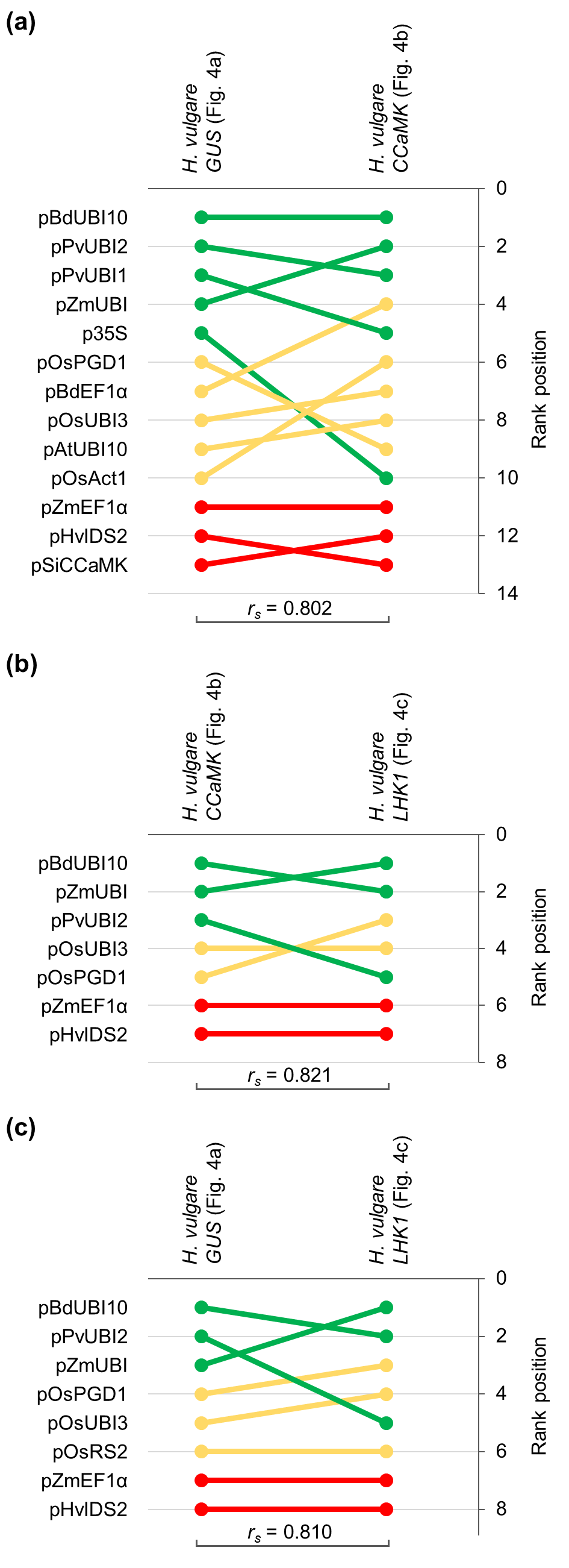

Supplement: Supplementary file 4 — Figure S4 Rank correlation analysis between promoter activities tested by GUS, CCaMK and LHK1 expression in barley. [file PBI-17-2234-s009.tif]

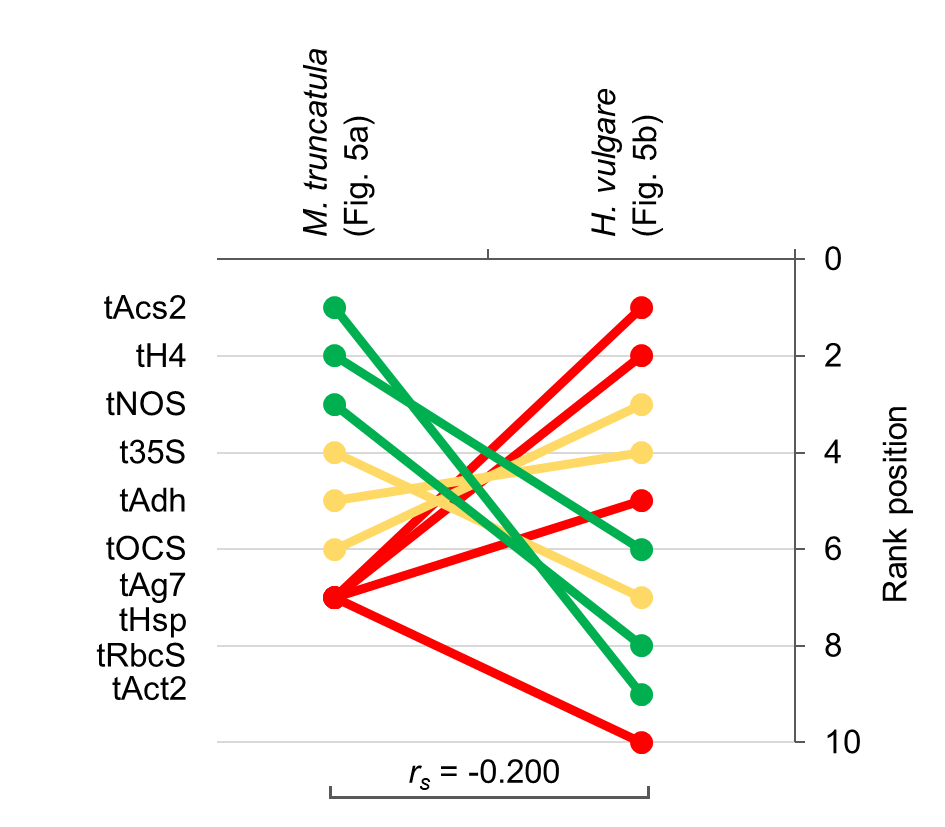

Supplement: Supplementary file 5 — Figure S5 Rank correlation analysis between terminator activities tested by ccamk mutant complementation in Medicago truncatula and LUC expression in barley. [file PBI-17-2234-s001.tif]

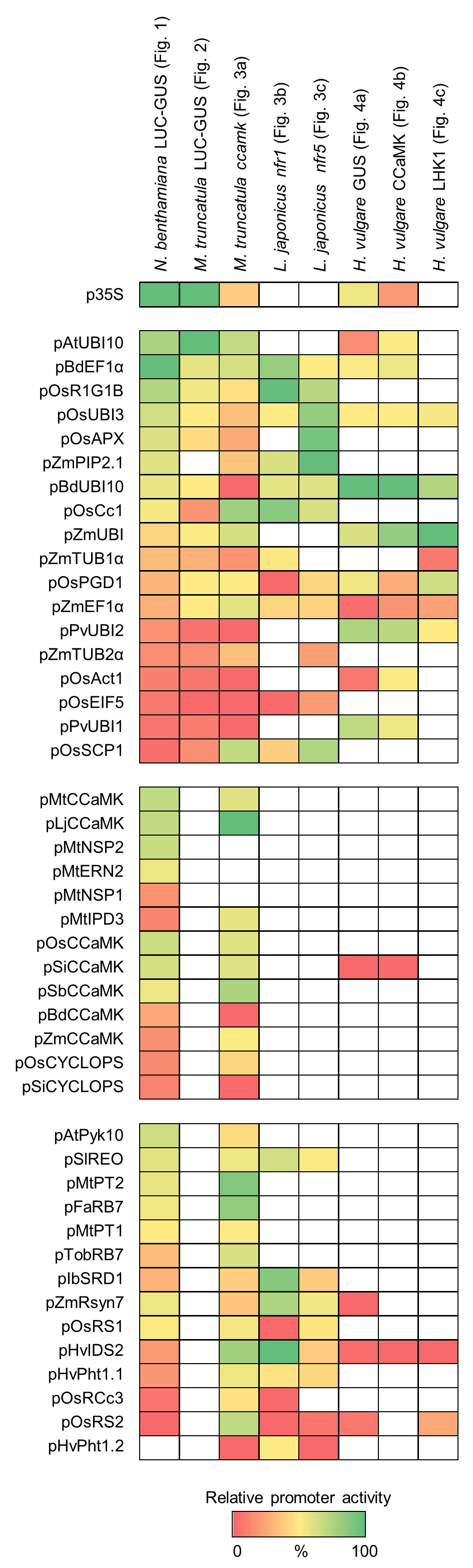

Supplement: Supplementary file 6 — Figure S6 Heat‐map summary of all promoter testing data. [file PBI-17-2234-s002.tif]
