# Supplementary material for: Characterizing standard genetic parts and establishing common principles for engineering legume and cereal roots
Source: Plant Biotechnol J. 2019 May 23;17(12):2234–45. doi: 10.1111/pbi.13135 (PMC6835126; doi:10.1111/pbi.13135)
Supplement: Supplementary file 7 — Table S1 Summary of GUS staining results in barley. Table S2 Primers used in this study. [file PBI-17-2234-s003.docx]

**SUPPORTING INFORMATION**

**Table S1: Summary of GUS staining results in barley.** GUS staining was performed on root material of 3 month old barley T0 plants grown on media containing hygromycin. For each promoter, 3-6 plants originating from independent transformation events were analysed. The hygromycin copy number of these plants varied between 1 and 3, but most plants were single copy lines. Blue shading indicates promoters for which GUS staining was positive in at least one of the analysed plants.

| **Promoter name** | **GUS positive/total** | **Subgroup** |
| --- | --- | --- |
| p35S | 3/4 | Constitutive |
| pZmUBI | 6/6 | Constitutive |
| pAtUBI10 | 6/7 | Constitutive |
| pOsCc1 | 0/3 | Constitutive |
| pOsSCP1 | 0/6 | Constitutive |
| pBdEF1α | 2/2 | Constitutive |
| pZmEF1α | 2/6 | Constitutive |
| pOsPGD1 | 3/6 | Constitutive |
| pOsR1G1B | 0/6 | Constitutive |
| pZmPIP2.1 | 0/6 | Constitutive |
| pOsUBI3 | 3/4 | Constitutive |
| pZmTUB2α | 0/6 | Constitutive |
| pZmTUB1α | 0/6 | Constitutive |
| pOsAPX | 0/6 | Constitutive |
| pOsEIF5 | 0/6 | Constitutive |
| pOsAct1 | 5/6 | Constitutive |
| pBdUBI10 | 6/6 | Constitutive |
| pPvUBI1 | 5/6 | Constitutive |
| pPvUBI2 | 5/5 | Constitutive |
| pLjCCaMK | 0/5 | Symbiosis-related |
| pMtCCaMK | 0/5 | Symbiosis-related |
| pSbCCaMK | 0/4 | Symbiosis-related |
| pSiCCaMK | 1/3 | Symbiosis-related |
| pOsCCaMK | 0/6 | Symbiosis-related |
| pZmCCaMK | 0/6 | Symbiosis-related |
| pBdCCaMK | 0/1 | Symbiosis-related |
| pOsCYCLOPS | 0/6 | Symbiosis-related |
| pSiCYCLOPS | 0/6 | Symbiosis-related |
| pMtIPD3 | 0/6 | Symbiosis-related |
| pMtNSP2 | 0/6 | Symbiosis-related |
| pMtNSP2 | 0/6 | Symbiosis-related |
| pMtERN2 | 0/6 | Symbiosis-related |
| pMtPT2 | 0/6 | Root-specific |
| pFaRB7 | 0/6 | Root-specific |
| pTobRB7 | 0/6 | Root-specific |
| pSlREO | 0/5 | Root-specific |
| pMtPT1 | 0/6 | Root-specific |
| pAtPyk10 | 0/5 | Root-specific |
| pIbSRD1 | 0/6 | Root-specific |
| pHvIDS2 | 2/5 | Root-specific |
| pOsRS2 | 1/6 | Root-specific |
| pHvPht1.1 | 0/6 | Root-specific |
| pOsRS1 | 0/6 | Root-specific |
| pOsRRc3 | 0/4 | Root-specific |
| pZmRsyn7 | 0/6 | Root-specific |

**Table S2: Primers used in this study.**

| **Name** | **Experiment/Gene/target** | **Sequence** |
| --- | --- | --- |
| GG1 | L0 vector backbone | caatacgcaaaccgcctc |
| GG2 | L0 vector backbone | CCTATAAAAATAGGCGTATCACG |
| GG3 | L1 & L2 vector backbone | cccgccaatatatcctgtc |
| GG4 | L1 & L2 vector backbone | GCGGACGTTTTTAATGTACTG |
| LUC_out_L | Cloning & colony PCR | TTCTTTATGTTTTTGGCAT |
| LUC_out_R | Cloning & colony PCR | CGGAAAGATCGCCGTGTAA |
| GUS_out_L | Cloning & colony PCR | ACATAAGGGACTGACCCAT |
| GUS_out_R | Cloning & colony PCR | AGCAGGGAGGCAAACAATGA |
| MtCCaMK_full_f | *CCaMK* amplification | atgggatatggaacaagaaaac |
| MtCCaMK_full_r | *CCaMK* amplification | ttatggacgaatagaagagagaac |
| sMtCCaMK_full_f | *sCCaMK* amplification | atgggctacggcacccgcaagct |
| sMtCCaMK_full_r | *sCCaMK* amplification | tcatggcctgatgctgctcagca |
| MtNSP2_full_f | *NSP2* amplification | ATGGATTTGATGGACATGG |
| MtNSP2_full_r | *NSP2* amplification | TAAATCAGAATCTGAAGAAG |
| GAPDH2_f | qRT-PCR barley *GAPDH2* | TCGATGAGGACCTTGTTTCC |
| GAPDH2_r | qRT-PCR barley *GAPDH2* | GCTGTATCCCCACTCGTTGT |
| sMtCCaMK_f | qRT-PCR *M. truncatula* *CCaMK* | AAGATCACCACCAAGAGCGA |
| sMtCCaMK_r | qRT-PCR *M. truncatula* *CCaMK* | TTGTTCTGGGCAATGAACGG |
| MtNSP2_f | qRT-PCR *M. truncatula* *NSP2* | GCTCGTTGGGCCGGATATAT |
| MtNSP2_r | qRT-PCR *M. truncatula* *NSP2* | TCACCGGAACTCCCCTAAAC |
| sMtNSP2_f | qRT-PCR *M. truncatula* *sNSP2* | AATGCAGAACAGGGCTAGGA |
| sMtNSP2_r | qRT-PCR *M. truncatula* *sNSP2* | CTCACCGCCAGTTCTGTAGA |
| LHK1_f | qRT-PCR *L. japonicus* *LHK1* | AAGTCCGAGTTCCACATGCC |
| LHK1_r | qRT-PCR *L. japonicus* *LHK1* | TAGCTGGCTTGGACTTGAAG |
| GUS_f | qRT-PCR *GUS* | CGACTGGGCAGATGAACATG |
| GUS_r | qRT-PCR *GUS* | CGCTTCGAAACCAATGCCTA |

**Figure S1: Rank correlation analysis between promoter activities tested by LUC-GUS assays in *Nicotiana benthamiana* and *Medicago truncatula*, and *ccamk* mutant complementation in *M. truncatula*.** Ranks for constitutive (a), symbiosis-related (b) and root-specific (c) promoters were derived according to experimental performance (1 = best promoter) and are plotted for each promoter and experiment as indicated. Colour coding denotes high (green), medium (yellow) or low (red) activity based on the promoter being ranked either in the top, middle or bottom third of all tested promoters in the *N. benthamiana* LUC-GUS assay. Spearman’s rank correlation coefficient (*r_s_*) is shown for pairwise comparisons between datasets. Strong correlation was observed between constitutive promoters tested in *N. benthamiana* and *M. truncatula* LUC-GUS assays (*r_s_* = 0.864, *p* = 3.85 x 10^-6^). Moderate correlation was observed between constitutive promoters tested in *M. truncatula* LUC-GUS assays and *M. truncatula ccamk* complementation (*r_s_* = 0.545, *p* = 0.019). No significant correlation was observed between constitutive promoters tested in *N. benthamiana* LUC-GUS assays and *M. truncatula ccamk* complementation (*r_s_* = 0.359, *p* = 0.143). Strong correlation was observed between symbiosis-related promoters tested in *N. benthamiana* LUC-GUS assays and *M. truncatula ccamk* complementation (*r_s_* = 0.794, *p* = 0.006). No correlation was observed between root-specific promoters tested in *N. benthamiana* LUC-GUS assays and *M. truncatula ccamk* complementation (*r_s_* = -0.101, *p* = 0.721).

**Figure S2: Rank correlation analysis between promoter activities tested by *ccamk* mutant complementation in *Medicago truncatula*, and *nfr1* and *nfr5* mutant complementation in *Lotus japonicus*.** Ranks for constitutive (a) and root-specific (b) promoters were derived according to experimental performance (1 = best promoter) and are plotted for each promoter and experiment as indicated. Colour coding denotes high (green), medium (yellow) or low (red) activity based on the promoter being ranked either in the top, middle or bottom third of all tested promoters in the *M. truncatula ccamk* mutant complementation experiment. Spearman’s rank correlation coefficient (*r_s_*) is shown for pairwise comparisons between datasets. No significant correlation was observed between constitutive promoters tested for complementation of *ccamk* and *nfr1* mutants (*r_s_* = 0.297, *p* = 0.405), *nfr1* and *nfr5* mutants (*r_s_* = 0.448, *p* = 0.194), or *ccamk* and *nfr5* mutants (*r_s_* = 0.073, *p* = 0.842). No significant correlation was observed between root-specific promoters tested for complementation of *ccamk* and *nfr1* mutants (*r_s_* = 0.167, *p* = 0.693), *nfr1* and *nfr5* mutants (*r_s_* = 0.179, *p* = 0.672), or *ccamk* and *nfr5* mutants (*r_s_* = -0.024, *p* = 0.955).

**Figure S3: GUS staining reveals that promoters from the standard genetic parts library show different levels of activity in barley*.*** Constructs containing promoters from the standard genetic parts library driving expression of the β-glucuronidase (*GUS*) reporter gene were stably expressed in barley via *Agrobacterium tumefaciens*-mediated transformation. Harvested chimeric roots were GUS stained overnight and representative images are shown for samples. Images are at 10x magnification.

**Figure S4: Rank correlation analysis between promoter activities tested by *GUS*, *CCaMK* and *LHK1* expression in barley.** Ranks for all promoters were derived according to experimental performance (1 = best promoter) and are plotted for each promoter and experiment as indicated. Comparisons are made between *GUS* and *CCaMK* (a), *CCaMK* and *LHK1* (b), and *GUS* and *LHK1* (c). Colour coding denotes high (green), medium (yellow) or low (red) activity based on the promoter being ranked either in the top, middle or bottom third of all tested promoters in the first assay of each panel. Spearman’s rank correlation coefficient (*r_s_*) is shown for pairwise comparisons between datasets. Strong correlation was observed between promoters tested for expression of *GUS* and *CCaMK* transgenes (*r_s_* = 0.802, *p* = 9.69 x 10^-4^), *CCaMK* and *LHK1* transgenes (*r_s_* = 0.821, *p* = 0.023), and *GUS* and *LHK1* transgenes (*r_s_* = 0.810, *p* = 0.015).

**Figure S5: Rank correlation analysis between terminator activities tested by *ccamk* mutant complementation in *Medicago truncatula* and *LUC* expression in barley.** Ranks for all terminators were derived according to experimental performance (1 = best terminator) and are plotted for each terminator and experiment as indicated. Colour coding denotes high (green), medium (yellow) or low (red) activity based on the terminator being ranked either in the top, middle or bottom third of all tested terminators in the *M. truncatula* assay. Spearman’s rank correlation coefficient (*r_s_*) is shown for pairwise comparison between datasets. No correlation was observed between terminators tested for *M. truncatula ccamk* complementation and expression of the *LUC* transgene in barley (*r_s_* = -0.200, *p* = 0.579).

**Figure S6: Heat-map summary of all promoter testing data.** All promoter testing data is presented as a heat-map where relative promoter activity (%) has been calculated for each experiment, setting the highest promoter activity for each experiment at 100% (see Supporting File 2 for data and % values). This summary gives a complete overview of the complexity of the data and variation between different promoters, plant species and tissue types. White cells indicate that no data was collect for a particular promoter under the given experimental condition.

**Supporting File 1: References and sequences of promoters, terminators and coding sequences used in this study.**

**Supporting File 2: Summary of all genetic part characterisation data presented in this study.**

**Supporting File 3: Summary of all Golden Gate constructs used in this study.**
